# Early diagnosis of sepsis due to Gram-negative infection with the Endotoxin Activity Assay

**DOI:** 10.1186/cc14077

**Published:** 2014-12-03

**Authors:** M Kang, M Tsunoda, M Saito, N Saito, M Namiki, M Takeda, T Harada, A Yaguchi

**Affiliations:** 1 Department of Critical Care and Emergency Medicine, Tokyo Women's Medical University, Tokyo, Japan

## Introduction

The Endotoxin Activity Assay (EAA™; Spectral Diagnostics Inc., Toronto, Canada) is a rapid *in vitro *diagnostic test of the neutrophil reaction to endotoxin and reflects the endotoxemia. A higher value of EAA (>0.60) has been shown to correlate with developing severe sepsis and a high mortality in other previous studies. We hypothesize that a value of EAA more than 0.55, not >0.60, may be useful to earlier diagnose sepsis due to Gram-negative organisms and to assess the severity.

## Methods

The present study is a single-center retrospective observational analysis of adult septic patients in whom EAA was performed from July 2008 to July 2013. Patients were divided into two groups: (1) EAA >0.55 and (2) EAA < 0.54. Age, sex, days of ICU stay, ICU mortality, body temperature, WBC, CRP, procalcitonin (PCT), SOFA score, and microbiological data were compared between two groups. Values are expressed as mean ± SD. Data were analyzed by chi-square test and Mann-Whitney U test. *P *< 0.05 was considered significant. The sensitivity, specificity, positive predictive value (PPV), negative predictive value (NPV) and odds ratio were also evaluated.

## Results

Five hundred and ninety-four patients (377 men and 217 women; mean age 65.0 ± 17.5 years) were studied. There were (1) 104 patients with EAA >0.55 and (2) 490 patients with EAA < 0.54. ICU mortality (39.4 vs. 26.6 %, *P *= 0.01), PCT (17.2 ± 36.0 vs. 11.8 ± vs. 30.6 ng/ml, *P *= 0.04), SOFA score (8.1 ± 4.9 vs. 6.2 ± 4.6, *P *= 0.04) and positive Gram-negative organisms in cultures (57.7 vs. 45.5 %, *P *= 0.02) were significantly higher in group (1) than group (2). Age, sex, body temperature, WBC and CRP were not significantly different between two groups. Using detections of Gram-negative organisms in cultures, the sensitivity, the specificity, the PPV and the NPV were 22.1, 85.2, 57.7 and 54.6%, respectively. The odds ratio was 1.64.

## Conclusion

ICU mortality and severity were higher in patients with EAA >0.55 than in patients with EAA < 0.54. There is a possibility that an EAA value more than 0.55 is a meaningful value for early diagnosis of sepsis due to Gram-negative infection.

